# Supplementary material for: Metabolic heterogeneity and niche rewiring in bone marrow plasma cells from patients with MGUS, solitary bone plasmacytoma and multiple myeloma
Source: Blood Cancer J. 2026 Jun 16;16(1):143. doi: 10.1038/s41408-026-01525-8 (PMC13507332; doi:10.1038/s41408-026-01525-8)
Supplement: Supplementary file 1 [file 41408_2026_1525_MOESM1_ESM.docx]

**Metabolic heterogeneity and niche rewiring in bone marrow plasma cells from patients with MGUS, solitary bone plasmacytoma and multiple myeloma**

Guoxing Zhang^1,2#^, Na Sun^1#^, Yogesh Chawla^3^, Dragan Jevremovic^4^, Taro Hitosugi^5^, Shaji K. Kumar^3^, Wilson I. Gonsalves^3*^, Axel Walch^1*^

^1^Research Unit Analytical Pathology, Helmholtz Zentrum München - German Research Center for Environmental Health, Neuherberg, Germany.

^2^Department of Colorectal Surgery, The First Affiliated Hospital, Fujian Medical University, Fuzhou, China.

^3^ Division of Hematology, Mayo Clinic, Rochester, Minnesota, USA.
^4^Department of Laboratory Medicine and Pathology, Mayo Clinic, Rochester, Minnesota, USA.

^5^Division of Oncology Research, Mayo Clinic, Rochester, Minnesota, USA.

^#^ These authors contributed equally to this work.

* Corresponding Authors

* Correspondence:

Wilson I. Gonsalves MD

Professor of Medicine

Division of Hematology

Mayo Clinic, Rochester, MN, USA

Phone: 507-284-2511
Email: Gonsalves.Wilson@mayo.edu

Prof. Dr. med. Axel Walch

Head, Research Unit Analytical Pathology

Phone: +49 89 3187-2739

Email: axelkarl.walch@helmholtz-munich.de

**Running Title:** **Spatial bone marrow metabolomics in MGUS, SBPmm and MM**

- **Supplemental Figure**
- **Supplemental Methods**
- **Supplemental References**
- **Supplemental Table**

**Supplemental Figure**

**Suppl. Figure 1. Early phosphorylated glycolytic intermediates relevant to FDG-PET in MGUS-like and MM**

Violin plots showing the distribution of Fructose 1,6-bisphosphate and Glucose 6-phosphate intensities in bone marrow tissue sections from patients with monoclonal gammopathy of undetermined significance (MGUS-like, n = 10) and multiple myeloma (MM, n = 10). Intensities were log-transformed as log10(intensity) prior to visualization. White dots indicate the median, with interquartile ranges shown as white bars. Group comparisons were performed using a two-sided Wilcoxon rank-sum test, and exact P values are indicated above each panel.

**Supplemental Methods**

*Patient Cohort*

Clinical residual bone marrow plasma and trephine bone marrow biopsy samples were obtained from patients undergoing bone marrow biopsy and aspiration as part of standard clinical evaluation at Mayo Clinic. Bone marrow plasma was isolated from whole bone marrow aspirates by centrifugation at 2400 × g for 10 min to separate the supernatant, followed by a second centrifugation under identical conditions. The resulting plasma was collected, stored in EDTA tubes, and frozen at −80 °C until analysis.

Relevant clinical and laboratory parameters—including bone marrow plasma cell percentage, S-phase fraction, cytogenetic abnormalities, and fluorescence in situ hybridization (FISH) findings—were abstracted from clinical records for analysis (**Table 1**). The proliferation score or S-phase % was derived by measuring the percentage of clonal plasma cells in the bone marrow aspirate of that biopsy sample that were found to be in the synthetic phase of the cell cycle division by multiparametric flow cytometry. Patient age and sex were recorded at the time of diagnosis; patient sex was recorded at sample collection. Due to limited sample size, analyses were not stratified by sex.

All eligible samples meeting inclusion criteria were included in the final analyses, with no patient or sample attrition. As this was a retrospective observational study, no randomization or intervention-based group allocation was performed.

*LC-MS assessment of bone marrow plasma*

Bone marrow plasma samples were stored at −80 °C and shipped on dry ice to Metabolon, Inc. (Morrisville, NC) for batch analysis. Samples were processed using the automated MicroLab STAR system (Hamilton Company). Recovery standards were added prior to extraction for quality control. Proteins were precipitated with methanol under vigorous shaking (2 min; GenoGrinder 2000, Glen Mills), followed by centrifugation to remove precipitated protein.

The resulting supernatants were dried using a TurboVap (Zymark) to remove organic solvent and stored overnight under nitrogen prior to analysis. Global untargeted metabolomic profiling was conducted by Metabolon, Inc. Extracts were analyzed by ultrahigh-performance liquid chromatography–tandem mass spectrometry (UPLC–MS/MS) using a Waters ACQUITY UPLC system coupled to a Thermo Scientific Q Exactive Orbitrap mass spectrometer equipped with a heated electrospray ionization (HESI-II) source. Analyses were performed in both positive (two methods) and negative (two methods) ion modes, with a mass resolution of 35,000 and a scan range of m/z 70–1000.

Metabolites were identified by automated comparison of ion features to a proprietary reference library of authenticated standards, followed by manual curation. Metabolite abundances were quantified by area-under-the-curve integration of chromatographic peaks.

*High-mass-resolution MALDI-FT-ICR-MSI analysis*

Archived FFPE bone-marrow biopsy specimens were obtained from Mayo Clinic as previously described^1^. Tissue sections (3 µm thick) were cut using a microtome (HM 355S, Microm; Thermo Fisher Scientific) and mounted onto indium–tin–oxide–coated glass slides (Bruker Daltonik, Bremen, Germany). Sections were incubated at 60 °C for 1 h, followed by deparaffinization in xylene (two washes, 8 min each).

A matrix solution of 9-aminoacridine hydrochloride monohydrate (10 mg/mL; Sigma-Aldrich, Germany) in 70% methanol was prepared and uniformly applied using a SunCollect automatic sprayer (Sunchrom, Friedrichsdorf, Germany). Matrix deposition consisted of eight spray layers, with flow rates of 10, 20, and 30 µL/min for the first three layers and 40 µL/min for the remaining five layers.

MALDI-MSI was performed using a Bruker Solarix 7 T FT-ICR mass spectrometer (Bruker Daltonik, Bremen, Germany) in negative ion mode. Each pixel was acquired with 100 laser shots at a frequency of 1000 Hz. Mass spectra were collected over an m/z range of 75–1000 with a spatial resolution of 50 µm.

Following MSI acquisition, matrix was removed by incubation in 70% ethanol for 10 min. Sections were then stained with hematoxylin and eosin (H&E) using a HistoCore SPECTRA ST multistainer (Leica, Wetzlar, Germany), coverslipped, and digitized using an AxioScan.Z1 slide scanner (Carl Zeiss) equipped with a 20× objective. Whole-slide images were visualized and exported in TIFF format using ZEN 3.12 Blue Edition software (Carl Zeiss).

*Data Acquisition and Processing*

Data acquisition and processing were performed as follows. MALDI-MSI data were processed using FlexImaging software (version 5.0, Bruker Daltonik) and SCiLS Lab software (version 2025b, Bruker Daltonik). Spectral intensities were normalized to the root mean square (RMS) of all acquired data points.

Peak annotation was conducted by accurate mass matching against reference entries in the Human Metabolome Database (HMDB) and the Kyoto Encyclopedia of Genes and Genomes (KEGG). Negative ionization adducts considered included M–H, M–H₂O–H, M+K–2H, M+Na–2H, and M+Cl, using a mass accuracy threshold of 4 ppm.

Annotated features corresponding to drugs, pesticides, plant-derived compounds, or other non-endogenous molecules were excluded from downstream analyses.

*Bisecting k-means clustering*

We applied bisecting k-means clustering to analyze spatial metabolomic heterogeneity in high-resolution MALDI-FT-ICR MSI data. Bisecting k-means is an unsupervised algorithm that iteratively divides a dataset by repeatedly applying k-means. It starts with all data points in one cluster and splits this cluster into two subclusters by maximizing inter-cluster differences. It then continues to bisect selected clusters until reaching the target number of clusters. We performed clustering in SCiLS Lab, which is specialized for mass spectrometry imaging analysis. To enrich tumor plasma cell regions and reduce confounding from non-cellular compartments, we restricted clustering to the bone marrow compartment and excluded bone trabeculae. We delineated the marrow region of interest on each section before clustering. This region-focused strategy increased the purity of plasma-cell-rich clusters. The approach effectively partitioned the metabolic landscape of FFPE bone marrow sections into biologically distinct regions. It reliably identified regions enriched for plasma cells in MGUS-like and plasmacytoma cells in MM. We further validated the accuracy and robustness of the clusters using dimensionality-reduction methods, which confirmed clear separation of precursor and malignant metabolic profiles across patient samples.

*Simpson’s diversity index as heterogeneity score*

The heterogeneity of cluster distribution among patients was assessed using Simpson’s diversity index, calculated by the following formula:

$$D = 1-\sum_{i=1}^{n} p_{i}^{2}$$

Here, represents the proportion of pixels assigned to cluster for each patient. This diversity index ranges from 0, indicating complete homogeneity (all pixels of a patient belong to one cluster), to 1, representing maximal heterogeneity (pixels evenly distributed across clusters). Thus, a higher value of reflects greater metabolic heterogeneity within the patient's clusters.

*Diversity and evenness analysis*

To quantitatively characterize metabolic heterogeneity within spatially defined tissue compartments, we employed diversity metrics derived from Hill numbers, a unified framework that expresses diversity as the effective number of equally abundant features and enables consistent comparison of abundance distributions across samples. Hill numbers generalize classical diversity indices, including species richness, Shannon entropy, and the inverse Simpson index, into a single parameterized family defined by the order q, and support formal partitioning of diversity into α, β, and γ components. Hill numbers of order q = 2 correspond to the inverse Simpson index and emphasize dominant features while down-weighting rare ones, making them well suited for characterizing systems with disproportionate feature contributions^2^.

Within this framework, evenness was used to quantify how uniformly metabolite abundances were distributed within each spatial compartment, independent of the total number of detected features. Evenness-based measures have also been applied in recent cancer studies as a quantitative approach to describe intratumoral heterogeneity and dominance patterns within tumor ecosystems^3^. Guided by this concept, we applied Hill-based evenness (*q* = 2) to assess the balance of metabolic programs within plasma-cell–rich regions and the surrounding microenvironment between MGUS-like and MM.

For each tissue section, clustered pixel compositions were summarized by the relative proportions $p_{i}$ of pixels assigned to each non-zero cluster within a given compartment. The Hill diversity of order *q* = 2 was calculated as:

$$D^{2}=\frac{1}{\sum_{i} p_{i}^{2}}$$

To obtain a unit-scaled evenness we used the Hill-based evenness

$$E_{2}=\frac{D^{2}}{k_{\text{eff}}}$$

where $k_{\text{eff}}$ is the number of non-zero clusters within that compartment (bounded to 0–1). For sensitivity and correlation analyses we also report Pielou evenness,

$$J=\frac{H}{\mathrm{lo}g S},\quad H=-\sum_{i} p_{i}\mathrm{lo}g p_{i}$$

With $S$ the number of non-zero clusters. Unless otherwise stated, “evenness” refers to the Hill-based $E_{2}$.

*Two-compartment (paired) β diversity with count-weighted α*

For each patient we quantified divergence between plasma-cell-rich and microenvironment compartments using multiplicative β within the Hill framework (*q*=2):

$$\beta=\frac{^{2}D\gamma}{{\alpha̅}_{w}}$$

Here, $^{2}D\gamma$ is computed after pooling pixel counts of the two compartments, and ${\alpha̅}_{w}$ is the count-weighted arithmetic mean of within-compartment diversities,

$${\alpha̅}_{w}=\frac{n_{\text{PCR}}\cdot{^{2}D}_{\text{PCR}}+n_{\text{ME}}\cdot{^{2}D}_{\text{ME}}}{n_{\text{PCR}}+n_{\text{ME}}}$$

with $n_{\text{PCR}}$ and $n_{\text{ME}}$the total pixel counts in the plasma-cell-rich and microenvironment compartments, respectively. This weighting preserves each compartment’s sampling depth and is mathematically consistent with Hill-number partitioning. Whole-ROI evenness (“global heterogeneity”) was computed by pooling the two compartments and applying the same $E_{2}$definition.

*Group comparisons and correlations*

Group differences in evenness $E_{2}$ and paired β were assessed using two-sided Mann–Whitney (Wilcoxon rank-sum) tests, accompanied by Cliff’s *δ* (95% CI) as an effect-size measure. For associations between evenness and bone-marrow plasma-cell percentage (PC%), we analyzed Pielou evenness $J$ to minimize dependence on $k_{\text{eff}}$. Because both PC% and $J$ lie on (0,1), values were clipped to (*ε*,1−*ε*) with *ε* =10^−6^ and transformed using the logit function. We report (i) non-parametric Spearman’s *ρ* and Kendall’s *τ* on the raw scale, (ii) Pearson’s *r* on logit-transformed variables, and (iii) ordinary least squares on the logit scale with HC3 robust standard errors (sandwich estimator) to mitigate heteroscedasticity. As a sensitivity analysis, we fitted an M-estimator robust regression (Huber loss). For visualization, fitted lines and 95% CIs from the logit model were back-transformed to the original PC% scale.

*Statistical analysis*

No formal power calculation was performed, as this study was retrospective and exploratory, using all available samples. No formal blinding was applied; however, data acquisition and downstream computational analyses relied on automated and unsupervised pipelines to minimize investigator bias.

Normality was assessed using the Shapiro–Wilk test, and homogeneity of variance was evaluated by Levene’s test. For two-group comparisons, two-sided Student’s t-tests were applied, with Welch’s correction used when variances were unequal. When data deviated from normality, the Mann–Whitney U (Wilcoxon rank-sum) test was used. Linear associations between continuous variables were evaluated using Pearson’s correlation coefficient (two-tailed), whereas Spearman’s rank correlation (ρ) was used for monotonic or rank-based relationships.

For metabolite–proliferation analyses (**Fig. 4A**), a curated set of 123 endogenous metabolites was correlated with per-patient proliferation scores within MSI-defined plasma-cell–rich regions using Pearson correlation, with MGUS-like and MM analyzed separately. Metabolite–proliferation associations were further assessed using simple linear regression models (y = β₀ + β₁x) (**Fig. 4C**), and regression slopes (β₁) were summarized at the metabolic pathway level to indicate positive or negative associations (**Fig. 4D**). Trend lines in scatter plots were generated using LOESS smoothing.

Unsupervised analyses included principal component analysis (PCA) for dimensionality reduction of metabolite data (**Fig. 6A,D**), with group separation in PCA space assessed by PERMANOVA. Spatial metabolite clustering (**Fig. 6B**) was performed using bisecting K-means clustering (K = 6) on MALDI mass spectra to identify intra-sample metabolic subregions. Metabolites driving cluster separation were ranked by variable importance in projection (VIP) scores derived from partial least squares discriminant analysis (PLS-DA) (**Fig. 6C**). In **Fig. 3B,** a CatBoost classifier was trained on metabolite profiles to distinguish sample groups, and SHAP values were used to rank feature importance.

Data are presented as mean ± s.d., with all individual data points shown unless otherwise noted.

**References**

1. Ly A, Buck A, Balluff B, Sun N, Gorzolka K, Feuchtinger A*, et al.* High-mass-resolution MALDI mass spectrometry imaging of metabolites from formalin-fixed paraffin-embedded tissue. Nat Protoc 11, 1428-43 (2016). <https://doi.org/10.1038/nprot.2016.081>.

2. Chao A, Chiu C-H, Jost L. Unifying Species Diversity, Phylogenetic Diversity, Functional Diversity, and Related Similarity and Differentiation Measures Through Hill Numbers. Annual Review of Ecology, Evolution, and Systematics 45, 297-324 (2014). <https://doi.org/https://doi.org/10.1146/annurev-ecolsys-120213-091540>.

3. Bouras E, Yu R, Kim AE, Markozannes G, Murphy N, Albanes D*, et al.* Using gene-environment interactions to explore pathways for colorectal cancer risk. eBioMedicine 121, 105964 (2025). <https://doi.org/https://doi.org/10.1016/j.ebiom.2025.105964>.

**Supplemental Table**

| Case Number | Solitary Bone Plasmacytoma Diagnosis | Progressed to MM | Time to Progression  (Month) |
| --- | --- | --- | --- |
| MGUS1 | No | N/A | N/A |
| MGUS2 | No | No | 80 |
| MGUS6 | No | No | 69 |
| MGUS7 | No | No | 76 |
| MGUS8 | No | No | 52 |
| MGUS12 | No | No | 59 |
| MGUS19 | No | N/A | N/A |
| SBPmm17 | Yes | Yes | 24 |
| SBPmm18 | Yes | Yes | 7 |
| SBPmm20 | Yes | Yes | 2 |

**Supplementary Table 1. Clinical follow-up and progression status of MGUS-like cases** Shown are the presence or absence of a history of solitary bone plasmacytoma, progression to multiple myeloma (MM), and time to progression or last available follow-up (in months). Progression to MM was observed exclusively among cases with a history of solitary bone plasmacytoma and minimal bone marrow involvement (SBPmm). For MGUS cases without SBPmm, no progression to MM was observed during the available follow-up period. N/A indicates that progression was not observed and/or follow-up information was not available at the time of analysis.
